# Atherosclerotic middle cerebral artery stenosis: adjacent arterial geometry, hemodynamics and plaque features

**DOI:** 10.1007/s11604-026-02041-2

**Published:** 2026-07-04

**Authors:** Yu Liu, Shuang Li, Yue Gu, Xuan Tian, Yuying Liu, Ziqi Li, Yinhei Li, Tingjun Liang, Haipeng Liu, Linfang Lan, Bonaventure Y. M. Ip, Jingwei Li, Hui Fang, Bo Song, Yuming Xu, Thomas W. H. Leung, Jia Liu, Xinyi Leng

**Affiliations:** 1 Shenzhen Institutes of Advanced Technology, Chinese Academy of Sciences, Shenzhen, China; 2 Department of Medicine and Therapeutics, The Chinese University of Hong Kong, Hong Kong, China; 3 Department of Neurology, Ren Ji Hospital, Shanghai Jiao Tong University School of Medicine, Shanghai, China; 4 Department of Geriatric Medicine, Nanjing Drum Tower Hospital, Affiliated Hospital of Nanjing University Medical School, Nanjing, China; 5 Research Centre for Intelligent Healthcare, Coventry University, Coventry, UK; 6 National Medical Research Association, Leicester, UK; 7 Cardiovascular Analytics Group, PowerHealth Research Institute, Hong Kong, China; 8 Department of Neurology, First Affiliated Hospital of Sun Yat-Sen University, Guangzhou, China; 9 Department of Neurology, Nanjing Drum Tower Hospital, Affiliated Hospital of Nanjing University Medical School, Nanjing, China; 10 Department of Neurology, First Affiliated Hospital of Zhengzhou University, Zhengzhou, China

**Keywords:** Intracranial atherosclerosis, Vessel geometry, Computational fluid dynamics, Hemodynamics, Wall shear stress

## Abstract

**Background:**

Adjacent vessel geometry may affect focal hemodynamics and plaque features in intracranial atherosclerotic disease.

**Purpose:**

To investigate the correlations of upstream (internal carotid artery (ICA) bifurcation) and focal arterial geometry of M1 middle cerebral artery (MCA-M1) with location of MCA-M1 plaques and focal hemodynamics, particularly wall shear stress (WSS) metrics.

**Methods:**

In patients with symptomatic atherosclerotic stenosis (50–99%) of MCA-M1, we assessed vessel geometry at ICA bifurcation and MCA-M1, including diameters and diameter ratios of different arterial segments, angles between arteries, and MCA-M1 tortuosity. The MCA-M1 plaque was defined as in proximal or distal halves of MCA-M1. Computational fluid dynamics (CFD) modeling was conducted to simulate adjacent blood flow and quantify relative wall shear stress (relative WSS, rWSS) across the MCA-M1 plaque (as relative to mean WSS at proximal normal segment). We investigated the associations of vessel geometry with the plaque location and rWSS metrics.

**Results:**

Among 132 patients (median age 62 years), smaller diameter of MCA-M1 origin (adjusted odds ratio = 0.19, 95%CI 0.08–0.43, *p* < 0.001), smaller diameter ratio of MCA-M1 origin and terminal (0.04, 0.01–0.17, *p* < 0.001), and larger MCA-M1 tortuosity (1.03, 1.00–1.05, *p* = 0.045) were associated with proximal (versus distal) MCA-M1 plaques, independent of patient characteristics. These geometric parameters were associated with higher rWSS and larger area of high-rWSS region, throughout the MCA-M1 plaque and in upstream and downstream plaque segments, in patients with proximal MCA-M1 plaques (most *p* < 0.05), but not in those with distal MCA-M1 plaques. CFD models also revealed different velocity profiles and flow patterns in cases with different ICA bifurcation and MCA-M1 geometry.

**Conclusions:**

Adjacent arterial geometry may affect plaque distribution in MCA-M1. Focal hemodynamics e.g., WSS and velocity profiles, may play a role underlying the associations.

**Supplementary Information:**

The online version contains supplementary material available at https://doi.org/10.1007/s11604-026-02041-2.

## Introduction

Intracranial atherosclerotic stenosis (ICAS) is an important cause of ischemic stroke and transient ischemic attack (TIA) around the world [1, 2]. Understanding how an ICAS lesion develops and evolves would help identify individuals possibly at a high risk of developing ICAS and subsequent stroke/TIA.

The effects of common vascular risk factors (e.g., hypertension, dyslipidemia) on the ICAS development and stroke occurrence have been well established. On the other hand, intrinsic factors such as the adjacent arterial geometry and consequent hemodynamics may also significantly affect the development and features of intracranial atherosclerotic plaques, which are usually geometrically focal lesions [3, 4]. For instance, previous studies found that the shape (straight, U-shaped, inverted U-shaped, S-shaped) and orientation (ventral-, dorsal-, superior- or inferior-oriented) of M1 middle cerebral artery (MCA-M1) were associated with plaque distribution in different quadrants of MCA-M1 cross-sections [5, 6]. Yet, the possible effects of upstream arterial geometry on MCA plaque features were seldom investigated. In addition, the adjacent hemodynamics could underlie the association between arterial geometry and plaque features. For instance, wall shear stress (WSS) could be affected by upstream and focal arterial geometry as indicated in previous carotid artery studies [7, 8], which has been proposed as a causative factor in the development of “high-risk” atherosclerotic plaques [9]. However, these were also understudied in ICAS.

In this study, we aimed to investigate the correlations of upstream (internal carotid artery (ICA) bifurcation) and focal (MCA-M1) arterial geometry with location of MCA-M1 plaques and focal hemodynamics (particularly WSS metrics).

## Materials and methods

### Study design and subjects

This was a cross-sectional substudy based on the Stroke Risk and Hemodynamics in Intracranial Atherosclerotic Disease (SOpHIA) study, a cohort study recruiting patients with ischemic stroke or TIA due to 50–99% ICAS confirmed by computed tomography angiography (CTA) at 3 teaching hospitals. SOpHIA study was approved by local clinical research ethics committee, and informed consent was provided by all patients.

Patients with symptomatic ICAS in MCA-M1, without ipsilateral A1 anterior cerebral artery (ACA-A1) hypoplasia, were analyzed in the current study. MCA-M1 segment was defined as the horizontal portion of the MCA, extending from its origin at the bifurcation of the ICA to its distal bifurcation or trifurcation into terminal branches within the Sylvian fissure. Those with other causes of intracranial stenosis (intracranial dissection, vasculitis, or moyamoya disease), in-stent restenosis, potential cardioembolic stroke, or tandem stenosis of extra- and intracranial arteries, were excluded. Demographics, vascular risk factors (e.g., hypertension, diabetes mellitus, dyslipidemia, and smoking) and laboratory test results (e.g., fasting glucose, glycated hemoglobin, triglyceride, high-density and low-density lipoprotein cholesterol) at baseline were collected.

### Assessment of vessel geometry at ICA bifurcation and MCA-M1 segment on CTA

CTA protocol was described previously [10, 11]. It was performed on a 64-slice CT scanner (Lightspeed VCT; GE Healthcare, Waukesha, WI, USA) in helical mode without tilting, covering from the skull base to the cortical regions well above the lateral ventricles. Images were acquired at 120 kVp, 550 mAs, 0.625 mm slice thickness, and 0.4 s rotation.

**Fig. 1:**
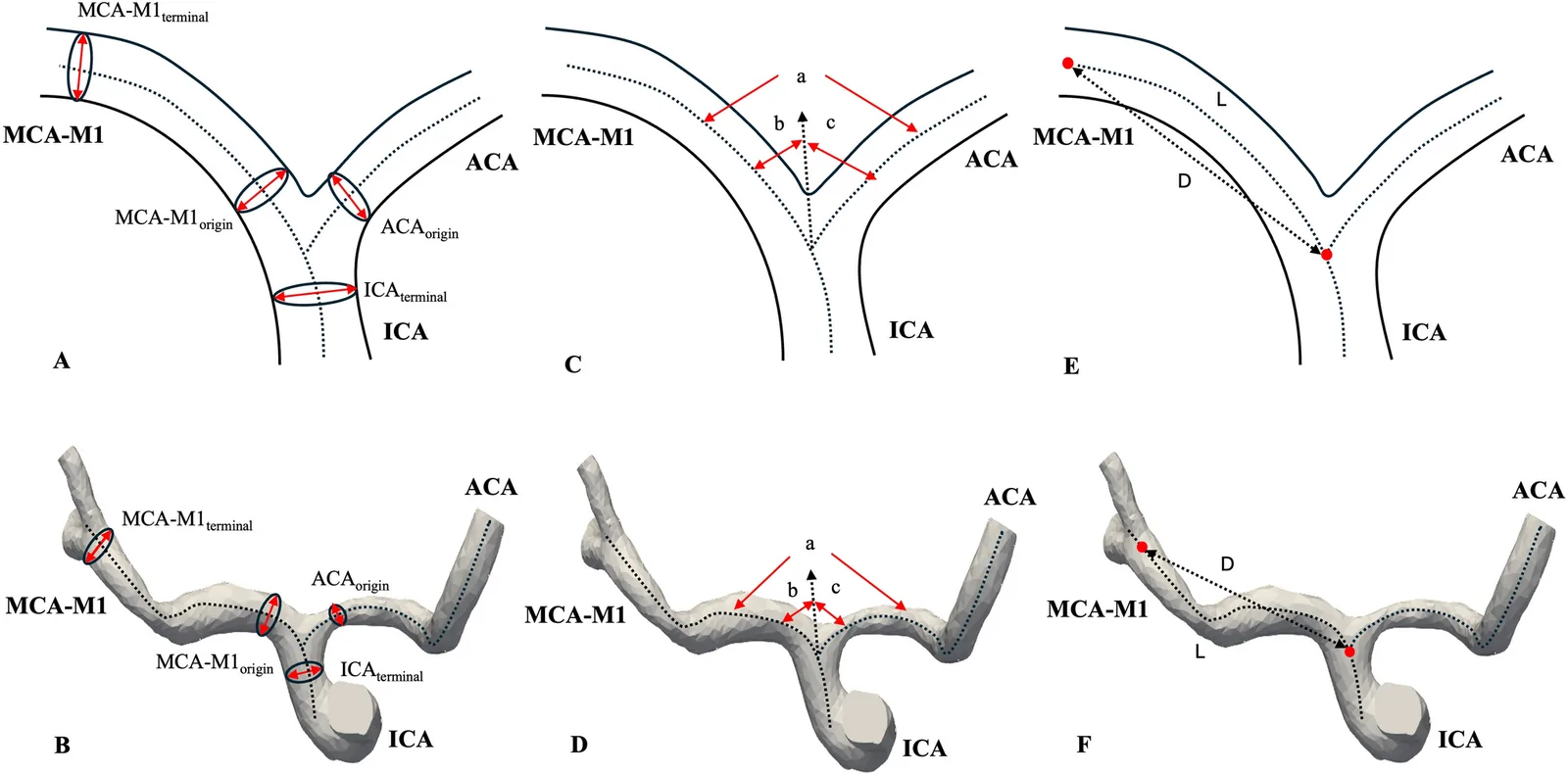
Schematic illustration and representative case of geometric feature assessment. **A**/**B** Measurement of diameters of ICA terminal, ACA origin, MCA-M1 origin and MCA-M1 terminal. **C**/**D** Measurement of angle between ACA and MCA-M1 (a), angle between ICA and MCA-M1 (b), angle between ICA and ACA **c**. **E**/**F** Measurement of MCA-M1 tortuosity. The actual length (L) of MCA-M1 segment and the straight-line distance (D) between MCA-M1 origin and terminal were used to calculate tortuosity as: MCA-M1 tortuosity = (L/D − 1) * 100%. Red markings indicate the locations and directions of measurement, and dotted lines indicate the vessel centrelines. ICA, internal carotid artery; ACA, anterior cerebral artery; MCA-M1, M1 middle cerebral artery

The geometric characteristics of ICA bifurcation and MCA-M1 segment were assessed on three-dimensional (3D) artery models reconstructed from CTA source images, using Mimics 21.0 software (Materialise, Ann arbor, MI), and were assessed by two investigators (Y.L. and Z.L.) with 5 and 4 years of relevant experience, respectively. Schematic illustrations and measurements on a representative case are provided in Fig. 1. First, arterial centerlines were extracted in Mimics and used as the geometric reference for subsequent measurements. The diameters of arterial segments (including ICA terminal, ACA origin, MCA-M1 origin and terminal; Fig. 1A/B) and angles between centerlines of these arterial segments were measured (Fig. 1C/D). The actual length of MCA-M1 and the straight-line distance between MCA-M1 origin and terminal were also measured, and the tortuosity of MCA-M1 segment was calculated by the relative difference between the actual length and the straight-line distance: (actual length/straight-line distance − 1) * 100% (Fig. 1E/F) [12]. The measurements showed substantial inter-rater reliability in 10 cases (Supplemental Table 5).

### Assessment of the MCA-M1 plaque on CTA

**Fig. 2:**
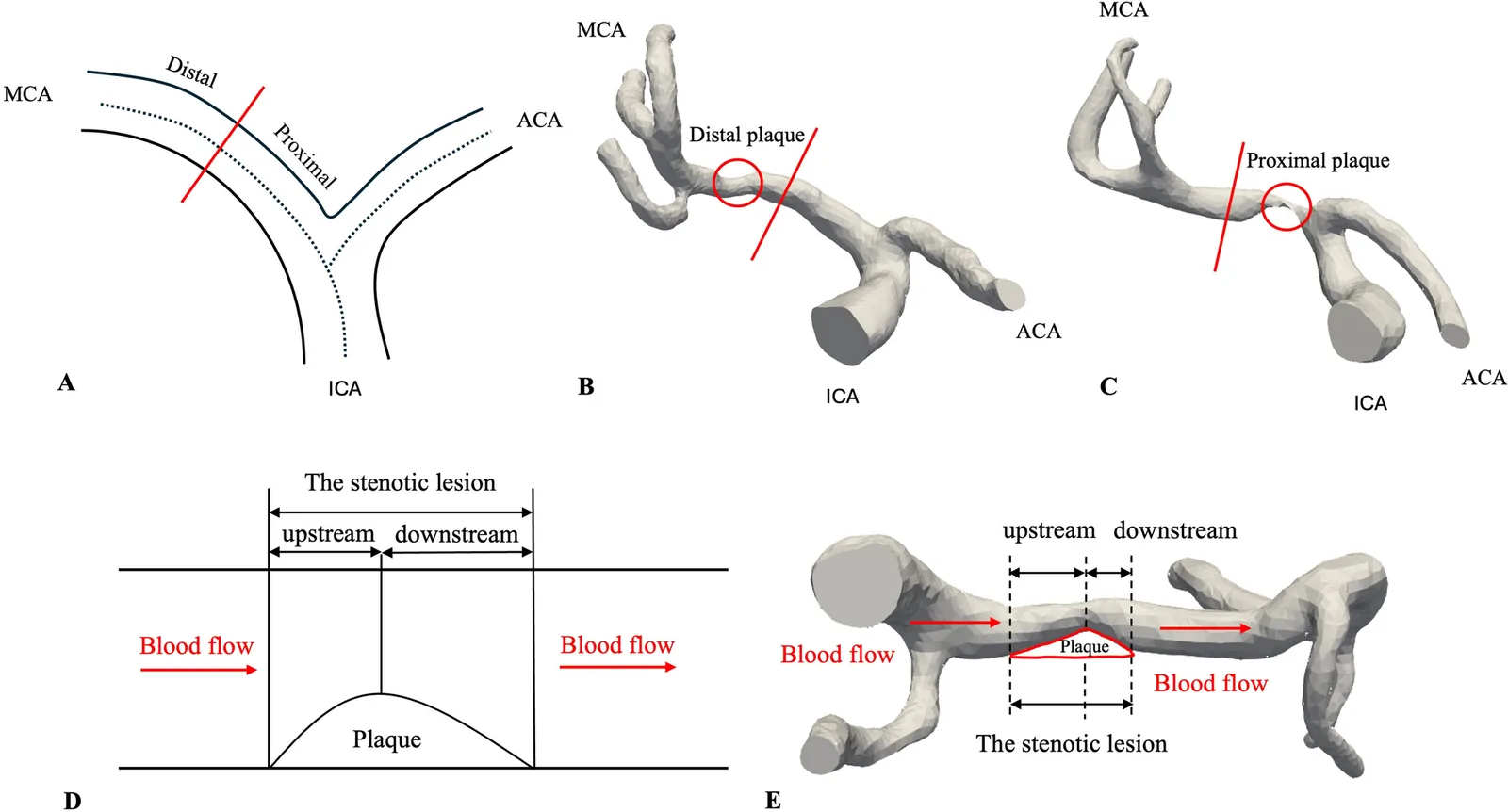
Schematic illustrations and representative cases for MCA-M1 plaque assessment. **A**–**C** Plaque location as being in the proximal or distal halves of MCA-M1. **D**/**E** Division of a plaque into upstream and downstream segments by the most severely narrowed site of the vessel lumen and the direction of blood flow. Red arrows indicate the blood flow direction. MCA-M1, M1 middle cerebral artery

The stenosis severity in MCA-M1 was measured on CTA images using the Warfarin-Aspirin Symptomatic Intracranial Disease (WASID) method, and classified as moderate (50–69%) or severe (70–99%) stenosis [13]. The plaque location was defined as in the proximal or distal halves of MCA-M1 (Fig. 2A), and was assessed by two investigators (Y.L. and S.L.) with 5 and 7 years of relevant experience, respectively. The measurements showed substantial inter-rater reliability in 10 cases (Supplemental Table 5). The plaque was further divided into the upstream and downstream segments, by the most severely narrowed site of the vessel lumen (Fig. 2B). Schematic illustrations and representative cases are provided in Fig. 2.

### Computational fluid dynamics (CFD) modeling and assessment of focal and global hemodynamic metrics

CFD modeling was conducted to simulate blood flow and quantify WSS metrics adjacent to MCA-M1 plaques. The modeling procedures were described in our previous work [10], including the following major steps: (i) 3D geometry of arteries was extracted from CTA source images, including intracranial ICA, MCA-M1, and ACA-A1 segments; (ii) Mesh was generated on the vessel wall and within the vessel lumen using ANSYS ICEM CFD (ANSYS version 15.0; ANSYS Inc., Canonsburg, PA, USA), with the maximal element sizes of 0.1 mm for inlet and outlets, and 0.25 mm for other parts of the model, containing 0.5 to 1 million elements in total for each case; (iii) Generic boundary conditions and blood properties were applied at the ICA inlet with a pressure of 110 mmHg, and at the MCA-M1 and ACA-A1 outlets with a mass flow rate based on mean flow velocities from a population-based study [14], using ANSYS CFX-Pre; rigid vessel wall with no-slip conditions were assumed, and blood was considered as an incompressible Newtonian fluid with a constant density of 1060 kgm^−3^ and viscosity of 0.0035 kg m^−1^ s^−1^; (iv) Steady-state blood flow simulation was conducted in ANSYS CFX by solving the Navier–Stokes equations; convergence was achieved when the root mean square residual value fell below 10^–4^.

WSS metrics were measured across the MCA-M1 plaques using ANSYS Ensight. To offset the effects of inter-subject variations on overall WSS values, relative WSS (rWSS) rather than absolute WSS values were analyzed as in our previous studies [11]. rWSS at any location across the plaque segment was defined as the ratio of absolute WSS at that location and mean WSS measured at a cross-section of distal ICA with normal vessel lumen and stable flow. rWSS > 3.0 was defined as high WSS [11]. We measured the maximum rWSS, area of high-rWSS region, and mean rWSS of high-rWSS region, throughout the MCA-M1 plaque and separately in the upstream and downstream plaque segments. The detailed methods were described in our previous study [11].

In addition, we also manually reversed the stenotic MCA-M1 to normal vessel lumen (with a same diameter with immediately proximal and distal arterial segments, or with a diameter smoothly tapering from the immediately proximal arterial segment) and conducted CFD modeling, to reveal the velocity profiles and flow patterns in a few cases with different geometric features at ICA bifurcation and MCA-M1. The findings would help explain the associations of vessel geometry, WSS distributions and MCA-M1 plaque features.

### Statistical analyses

Continuous variables were summarized by medians (interquartile range, IQR) and categorical variables by numbers (%). Demographics, vascular risk factors, laboratory test results, and arterial geometric characteristics, were compared between patients with different plaque locations (proximal and distal MCA-M1 plaques), using Mann–Whitney *U* tests for continuous variables and Pearson’s chi-square tests for categorical variables. Each geometric feature with *p* < 0.10 in univariate comparison was then analyzed in multivariate logistic regression, adjusting for systemic factors with *p* < 0.10 in univariate comparisons above, for its independent relationship with MCA-M1 plaque location. Adjusted odds ratios (aOR) and the confidence interval (CI) were obtained. The geometric features were not simultaneously included in one multivariate logistic regression model due to significant multicollinearity. Additionally, univariate comparisons for the differences in the geometric features between those with proximal and distal MCA-M1 plaques were also conducted separately in moderate and severe stenosis subgroups. The correlations of geometric characteristics with the rWSS metrics were separately analyzed in patients with proximal and distal MCA-M1 plaques, using Spearman’s correlation coefficient. All data were analyzed using the IBM SPSS statistics version 29.0 (IBM Corp., Armonk, NY, USA). Two-tailed *p* < 0.05 was considered statistically significant.

## Results

**Fig. 3:**
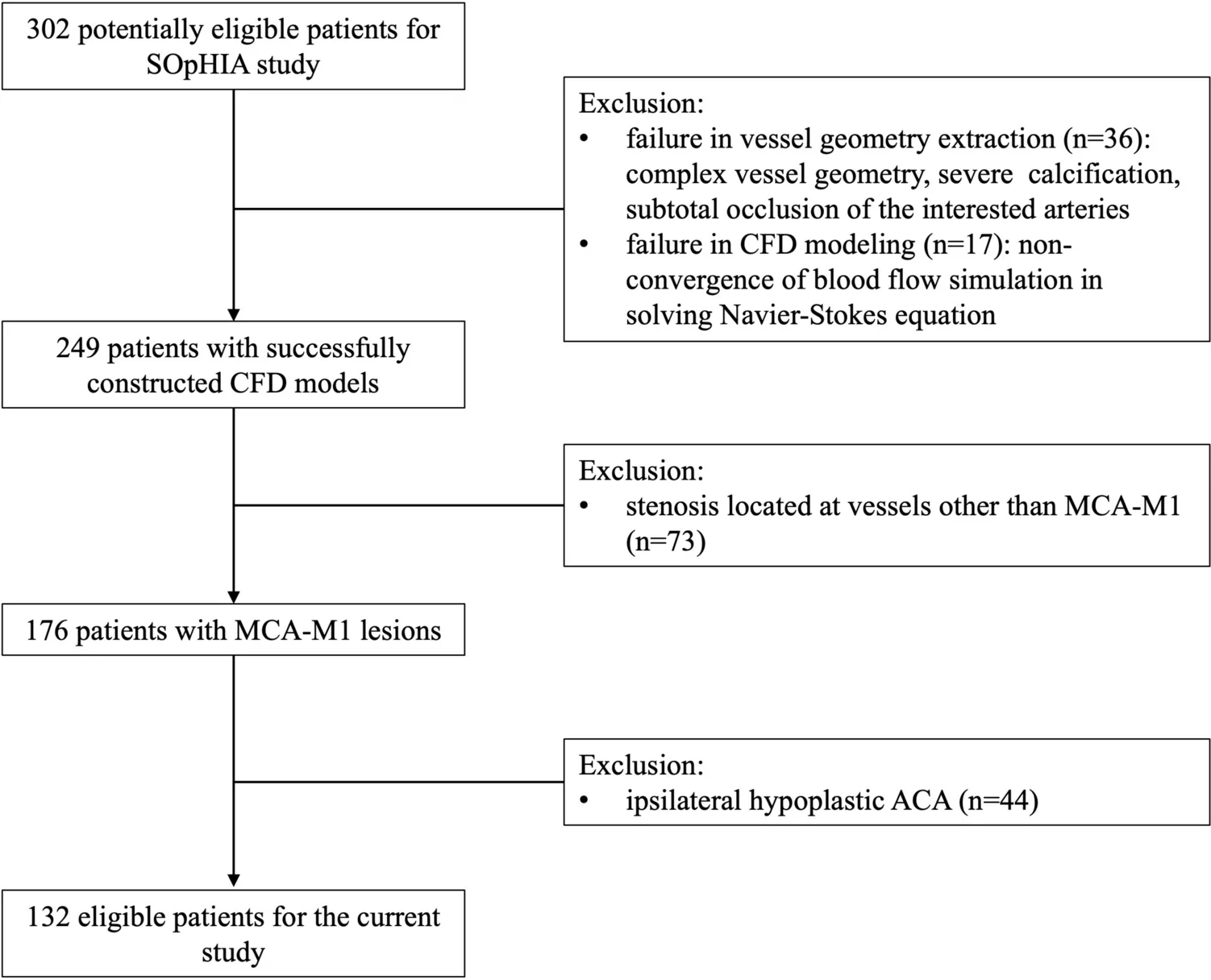
Flowchart of patient screening from SOpHIA study. SOpHIA, Stroke Risk and Hemodynamics in Intracranial Atherosclerotic Disease study; CFD, computational fluid dynamics; MCA-M1, M1 middle cerebral artery; ACA, anterior cerebral artery

The flowchart of patient screening from the SOpHIA cohort is shown in Fig. 3. In total, 132 patients (median age 62 years) with symptomatic, atherosclerotic MCA-M1 stenosis were analyzed. Of these patients, 86 (65.2%) had proximal MCA-M1 plaques and 46 (34.8%) had distal plaques; 74 (56.1%) patients had moderate (50–69%) stenosis and 58 (43.9%) had severe (70–99%) stenosis. Characteristics of the patients and the MCA-M1 plaques are reported in Table 1.

**Table 1:** Characteristics of the patients and the MCA-M1 plaques

Patient and plaque characteristics	N = 132
Age, year	62 (53–69)
Male	93 (70.5)
SBP at admission, mmHg	154 (135–169)
DBP at admission, mmHg	82 (76–95)
NIHSS	2 (1–4)
*Vascular risk factors*	
Hypertension	83 (62.9)
Diabetes mellitus	41 (31.1)
Dyslipidemia	75 (56.8)
Smoking	56 (42.4)
Previous stroke/TIA	19 (14.4)
*Laboratory test results*	
Fasting glucose, mmol/L	5.5 (4.9–7.0)
HbA1c, %	6.1 (5.6–6.9)
Triglyceride, mmol/L	1.4 (1.1–1.9)
HDL-C, mmol/L	1.1 (1.0–1.4)
LDL-C, mmol/L	3.0 (2.2–3.9)
*MCA-M1 plaque characteristics*	
Plaque location	
Proximal MCA-M1 plaque	86 (65.2)
Distal MCA-M1 plaque	46 (34.8)
Stenotic severity	
Moderate (50–69%) stenosis	74 (56.1)
Severe (70–99%) stenosis	58 (43.9)

Values are medians (interquartile range) or numbers (%)

*MCA-M1* M1 middle cerebral artery, *SBP* systolic blood pressure, *DBP* diastolic blood pressure, *NIHSS* National Institute of Health Stroke Scale, *TIA* transient ischemic attack, *HbA1c* glycated hemoglobin, *HDL-C* high-density lipoprotein cholesterol, *LDL-C* low-density lipoprotein cholesterol

### Association between vessel geometry and MCA-M1 plaque location

Supplemental Table 1 shows the patient and vessel geometry characteristics of those with proximal versus distal MCA-M1 plaques. Among the systemic factors (demographics, vascular risk factors and laboratory test results), only male sex (*p* = 0.066) and glycated hemoglobin (HbA1c) (*p* = 0.095) tended to differ between the two groups. Of the vessel geometry features, diameters of ICA terminal (3.60 vs. 3.70, *p* = 0.098) and MCA-M1 origin (2.60 vs. 3.14, *p* < 0.001), and diameter ratio of MCA-M1 origin and terminal (0.98 vs. 1.19, *p* < 0.001) were smaller in patients with proximal than distal MCA-M1 plaques. The MCA-M1 tortuosity was higher (20.48 vs. 13.64, *p* = 0.025) in patients with proximal than distal MCA-M1 plaques. In multivariate logistic regression (Table 2), a smaller diameter of MCA-M1 origin (aOR = 0.19, 95%CI = 0.08–0.43, *p* < 0.001), a smaller diameter ratio of MCA-M1 origin and terminal (aOR = 0.04, 95%CI = 0.01–0.17, *p* < 0.001), and higher MCA-M1 tortuosity (aOR = 1.03, 95%CI = 1.00–1.05, *p* = 0.045) were independently associated with proximal (versus distal) MCA-M1 plaques, after adjusting for sex and HbA1c.

**Table 2:** Multivariate logistic regression for the independent associations between vessel geometry characteristics and proximal versus distal MCA-M1 plaques

Geometric characteristics	Adjusted OR (95% CI)*	*P* value
Diameter of ICA terminal, mm	0.54 (0.26–1.11)	0.094
Diameter of MCA-M1 origin, mm	0.19 (0.08–0.43)	< 0.001
Diameter ratio of MCA-M1 origin and terminal	0.04 (0.01–0.17)	< 0.001
MCA-M1 tortuosity, %	1.03 (1.00–1.05)	0.045

*Adjusted for sex and glycated hemoglobin (HbA1c)

*MCA-M1* M1 middle cerebral artery, *ICA* internal carotid artery, *OR* odds ratio, *CI* confidence interval, *HbA1c* glycated hemoglobin

### Association between vessel geometry and MCA-M1 plaque location in moderate/severe MCA-M1 stenosis

Among the 74 patients with moderate MCA-M1 stenosis, 48 had proximal MCA-M1 plaques and 26 had distal plaques. Among the 58 patients with severe stenosis, 38 had proximal MCA-M1 plaques and 20 had distal plaques (Supplemental Table 2). There was no significant difference in the systemic factors between patients with proximal and distal MCA-M1 plaques in either moderate or severe stenosis subgroups. The diameter of MCA-M1 origin and the diameter ratio of MCA-M1 origin and terminal were significantly smaller in patients with proximal than distal MCA-M1 plaques, in both moderate and severe stenosis subgroups (*p* < 0.05), consistent with the overall analyses above. Yet, the higher MCA-M1 tortuosity in those with proximal than distal MCA-M1 plaques observed in the overall analyses above, was observed in the severe stenosis subgroup (23.46 vs. 11.11, *p* = 0.013) but not in the moderate stenosis subgroup.

### Comparisons of WSS metrics between proximal and distal MCA-M1 plaques

Table 3 shows the comparisons of rWSS metrics between proximal and distal MCA-M1 plaques. In upstream plaque segment, the area of high‑rWSS region was significantly larger in proximal than distal MCA-M1 plaques (0.54 vs. 0.40, *p* = 0.022). Other rWSS metrics were not significantly different between proximal and distal MCA-M1 plaques, when comparing the entire MCA-M1 plaque or separately in upstream/downstream MCA-M1 plaque segments*.*

**Table 3:** Comparison of rWSS metrics between proximal and distal MCA-M1 plaques

	rWSS metrics	Proximal MCA-M1 plaque	Distal MCA-M1 plaque	*P* value
Throughout the MCA-M1 plaque	Maximum rWSS	13.43 (7.22–19.49)	11.71 (6.54–19.95)	0.414
	Area of high-rWSS region, mm^2^	0.56 (0.36–0.71)	0.46 (0.33–0.60)	0.078
	Mean rWSS of high-rWSS region	6.31 (4.55–8.52)	5.54 (4.17–8.63)	0.259
Upstream MCA-M1 plaque segment	Maximum rWSS	11.79 (6.90–18.56)	10.08 (5.85–19.24)	0.372
	Area of high-rWSS region, mm^2^	0.54 (0.35–0.83)	0.40 (0.24–0.61)	0.022
	Mean rWSS of high-rWSS region	5.47 (4.30–8.31)	5.58 (3.82–8.98)	0.591
Downstream MCA-M1 plaque segment	Maximum rWSS	13.20 (7.08–19.05)	9.60 (6.30–19.20)	0.403
	Area of high-rWSS region, mm^2^	0.52 (0.35–0.63)	0.56 (0.34–0.68)	0.513
	Mean rWSS of high-rWSS region	6.77 (4.51–9.32)	5.60 (4.12–8.82)	0.477

*rWSS* relative wall shear stress, *MCA-M1* M1 middle cerebral artery

### Associations between vessel geometry and WSS metrics

The associations between vessel geometry and WSS metrics throughout the MCA-M1 plaque, and separately in the upstream and downstream plaque segments, in patients with proximal and distal MCA-M1 plaques, were presented in Supplemental Tables 3 and 4, respectively.

In patients with proximal MCA-M1 plaques (Supplemental Table 3), a smaller diameter of MCA-M1 origin, smaller diameter ratio of MCA-M1 origin and terminal, and a larger angle between ICA and MCA-M1 were associated with higher maximum rWSS, larger area of high-rWSS region, and higher mean rWSS of high-rWSS region throughout the MCA-M1 plaque (most *p* for Spearman correlation analyses < 0.05). The diameter of MCA-M1 origin and the angle between ICA and MCA-M1 also showed correlations with high rWSS metrics with the same trend in the upstream and downstream plaque segments, while the diameter ratio of MCA-M1 origin and terminal only correlated with rWSS metrics in the upstream plaque segment but not in the downstream segment.

Of note, there was no significant association between the vessel geometry features and the rWSS metrics, throughout the MCA-M1 plaque and separately in the upstream and downstream plaque segments, in patients with distal MCA-M1 plaques (Supplemental Table 4).

### Differences in velocity profiles and flow patterns by vessel geometric features

**Fig. 4:**
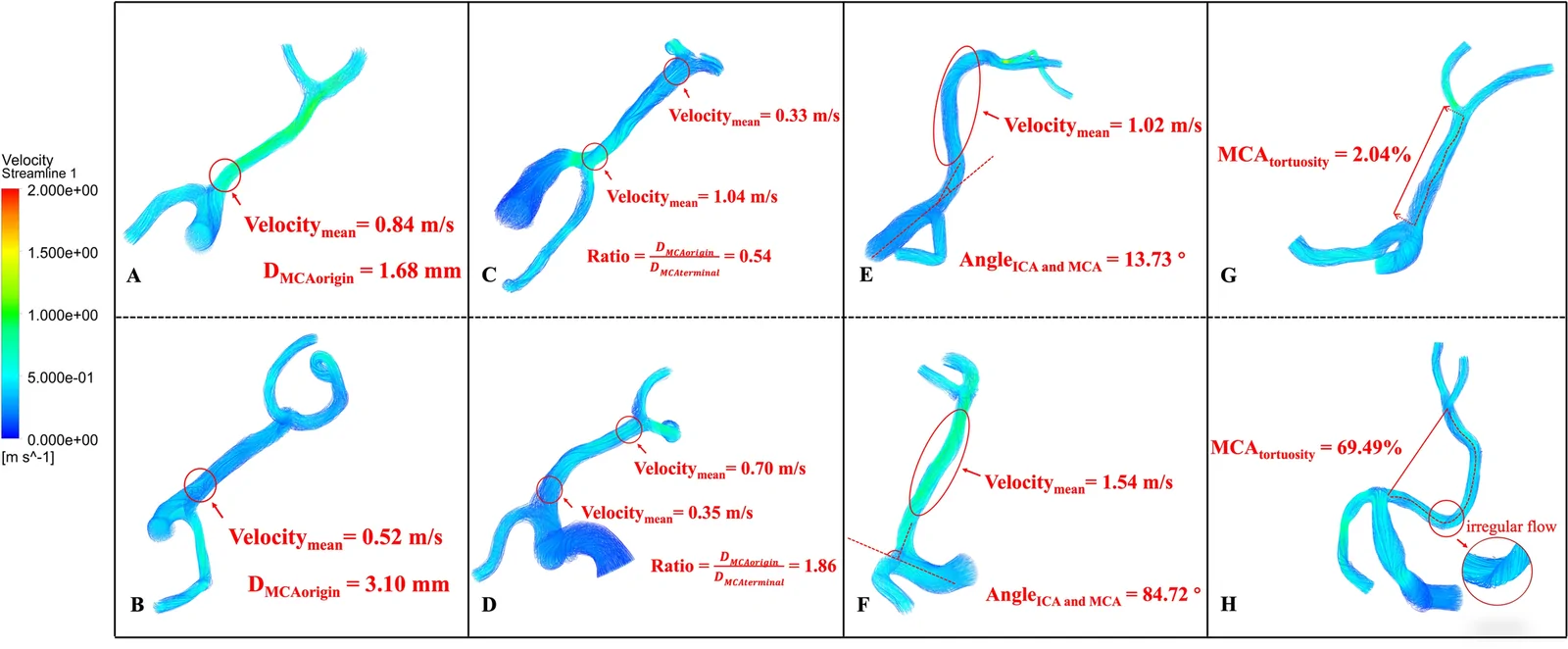
CFD models with manually restored normal vessel lumen, illustrating the different velocity profiles and/or flow patterns in 8 cases with different geometric features at ICA bifurcation and MCA-M1. **A**/**B** Higher flow velocity across MCA-M1 origin (0.84 m/s vs. 0.52 m/s), in a patient with a smaller MAC-M1 origin diameter (1.68 mm, Case A) compared to a patient with a larger MCA-M1 origin diameter (3.10 mm, Case B). **C**/**D** Different patterns of flow velocities from MCA-M1 origin to terminal, in a patient with a smaller (0.54, Case C) and a patient with a larger (1.86, Case D) diameter ratio of MCA-M1 origin and terminal. The mean velocity decreased from 1.16 m/s at the MCA-M1 origin to 0.39 m/s at the MCA-M1 terminal in Case C, which increased from 0.35 m/s at the MCA-M1 origin to 0.70 m/s at the MCA-M1 terminal in Case D. **E**/**F** Higher mean flow velocities along the entire MCA-M1 segment (1.54 m/s vs. 1.02 m/s), in a patient with a larger ICA and MCA-M1 angle (84.72°, Case F) compared to a patient with a smaller ICA and MCA-M1 angle (13.73°, Case E). **G**/**H** No obvious irregular flow in a MCA-M1 with tortuosity of 2.04% (Case G); irregular flow (solid arrow) observed in a curved MCA-M1 with tortuosity of 69.49% (Case H). CFD, computational fluid dynamics; ICA, internal carotid artery; MCA-M1, M1 middle cerebral artery

We manually reversed the stenotic MCA-M1 to normal vessel lumen and conducted CFD modeling, in 8 cases with different geometry in adjacent arterial segments and different MCA-M1 tortuosity, which showed different velocity profiles and flow patterns (Fig. 4). For instance, flow velocity across the MCA-M1 origin was higher with a smaller MCA-M1 origin diameter (Cases A/B). There was decreasing flow velocity from MCA-M1 origin to terminal with a smaller MCA-M1 origin and terminal diameter ratio, but increasing flow velocity from MCA-M1 origin to terminal with a larger MCA-M1 origin and terminal diameter ratio (Cases C/D). Moreover, there were higher mean flow velocities across the MCA-M1 segment with a larger ICA and MCA-M1 angle (Case E/F). In addition, turbulent flow was more likely to be observed in curved MCA-M1 with a higher tortuosity but not in a straight MCA-M1 (Cases G/H).

## Discussion

In this study of patients with symptomatic atherosclerotic MCA-M1 stenosis, we found significant associations of the geometric characteristics of ICA bifurcation and MCA-M1 with plaque location in MCA-M1 and high WSS distributions across the plaques. Specifically, patients with smaller diameter of MCA-M1 origin, smaller diameter ratio of MCA-M1 origin and terminal, and larger MCA-M1 tortuosity were more likely to have proximal than distal MCA-M1 plaques, which were mostly consistent in subgroups of patients with moderate and severe MCA-M1 stenosis. Smaller diameter of MCA-M1 origin, smaller diameter ratio of MCA-M1 origin and terminal, and larger angle between ICA and MCA-M1 were mostly associated with higher WSS and larger area of high-WSS regions throughout the MCA-M1 plaque and in the upstream and downstream plaque segments, in patients with proximal MCA-M1 plaques, but not in those with distal MCA-M1 plaques. CFD models also revealed different velocity profiles and flow patterns in cases with differences in these geometric features.

This study provides an integrated assessment of arterial geometry adjacent to symptomatic, atherosclerotic MCA-M1 stenosis, including that of the terminal ICA, ICA bifurcation, MCA-M1 and ACA-A1, of which some characteristics showed close associations with plaque distribution and focal hemodynamics. The findings echoed previous findings that MCA morphology was associated with MCA plaque distribution [5]. Meanwhile, the current analyses may provide additional insights by including upstream ICA geometry in the analysis, allowing a more comprehensive evaluation of the vascular configuration in association with plaque location and focal hemodynamic alterations, as terminal ICA constitutes the proximal inflow pathway to MCA-M1. Moreover, the finding that the geometric features at these arterial segments were associated with rWSS only in the proximal MCA-M1 plaque group but not in the distal MCA-M1 plaque group was seldom reported in previous studies, which is further discussed below.

The arterial geometric characteristics are relatively stable over time, which most likely influence plaque development at the plaque-free or early formation stage. Prior imaging studies have shown that baseline vascular geometry affects flow separation, WSS distribution, and regional susceptibility to atherosclerosis in both extracranial and intracranial arteries [5, 15–17]. Our CFD simulations (Fig. 4), performed on virtually restored lumen models, further illustrate how these geometric traits shape the velocity field independent of stenosis. Across the models, smaller M1 origin diameter, more abrupt proximal-to-distal caliber transitions, and wider ICA-MCA angles consistently produced higher or more uneven velocity distributions. In addition, highly tortuous vessels generated more irregular and unstable flow patterns, with rapid changes in flow direction along the lumen, which are features indicative of disturbed flow and linked to unfavorable endothelial responses and increased vulnerability to atherogenesis [18, 19]. These results support the concept that vessel geometry creates a pre-existing hemodynamic environment, predisposing certain MCA-M1 regions, particularly proximal segments, to higher or more variable WSS distributions associated with atherogenesis. However, the WSS measurements in our study were derived from CTA acquired after a symptomatic ischemic event, reflecting the hemodynamics of established and stenotic plaques. The high WSS and large high-WSS areas observed in proximal plaques likely represent the post-stenotic hemodynamic consequences of both local geometry and plaque morphology, rather than the WSS conditions at plaque initiation. This aligns with previous literature suggesting that low and variable WSS promotes plaque formation, while long-term high WSS contributes to plaque remodeling, vulnerability or subsequently rupture at later stages of atherosclerosis [4]. Taken together, our findings indicate that adjacent arterial geometry mainly influences initial plaque formation sites, while the WSS patterns in the stenotic state may affect subsequent plaque behavior. Of note, the finding that distal ICA bifurcation geometry was associated with WSS measures only in proximal MCA-M1 plaques but not in distal MCA-M1 plaques suggests that the hemodynamic alterations in proximal MCA-M1 are more directly exposed to the influence of terminal ICA and proximal MCA-M1 configuration, while hemodynamics in distal MCA-M1 may be more affected by the arterial geometry of distal MCA-M1 and beyond.

The current findings have implications for imaging assessment and risk stratification in symptomatic ICAS. First, adjacent arterial geometry may serve as a practical imaging biomarker that complements common vascular risk factors, stenosis severity and plaque characteristics, in the assessment of ICAS lesions and affected patients. Previous studies have associated MCA geometric features with plaque distribution and stroke risk [5], and associated CFD- or 4D flow MRI-derived WSS metrics with stroke mechanisms, plaque morphology, stroke recurrence, and change in the stenotic severity [10, 11, 20, 21]. In this context, integrating adjacent arterial geometric measures with plaque morphology and vascular remodeling patterns may help identify plaques residing in high-risk geometric and hemodynamic environments. Further longitudinal studies are warranted to verify the value of these geometric and hemodynamic features in predicting plaque progression, recurrent ischemic stroke, and treatment response. Second, the associations between simple geometric metrics and WSS distributions support the development of geometry-informed surrogate models for hemodynamic assessment. Although previous studies have shown the prognostic value of CFD based on routine CTA/MRA for stroke recurrence in sICAS [10, 22], wide clinical application remains limited by the computational demands and workflow complexity. To overcome this, data-driven surrogate models, such as simplified analytic formulas or machine-learning models trained on CFD datasets, could estimate key hemodynamic parameters directly from vessel geometry. Such approaches would enable efficient, scalable hemodynamic assessment without case-by-case simulation and facilitate its integration into clinical workflows [23, 24].

There were limitations of this study. First, this study had a relatively small sample size and was limited in patients with symptomatic ICAS in MCA-M1 but no other arterial segments, which may limit the generalizability of our findings. Moreover, significant associations of arterial geometry and focal hemodynamics were identified only for proximal MCA-M1 plaques but not for distal MCA-M1 plaques. Therefore, larger-scale studies involving various ICAS locations are warranted to confirm these results. Second, we conducted static CFD modeling, which can not reveal WSS oscillation and changes of the flow dynamics within and across cardiac cycles, which may also help explain the associations of vessel geometry and plaque features. Third, without vessel wall imaging, we did not assess detailed plaque characteristics such as plaque burden, vascular remodeling or contrast enhancement. Future studies incorporating vessel wall imaging and transient CFD modeling could enable a more detailed analysis over the associations of arterial geometry, flow dynamics and plaque morphology and composition. Finally, this was a cross-sectional study that cannot reflect the continuous changes of plaque and hemodynamics and their interactions, which need to be investigated in longitudinal studies.

## Conclusions

Geometric characteristics of ICA bifurcation and MCA-M1 segment may affect plaque distribution in MCA-M1. Focal hemodynamic patterns, such as high WSS distributions and velocity profiles, may underlie the associations.

## Supplementary Information

Below is the link to the electronic supplementary material.

Supplementary file1 (DOCX 50 KB)
