# Supplementary material for: Atherosclerotic middle cerebral artery stenosis: adjacent arterial geometry, hemodynamics and plaque features
Source: Jpn J Radiol. 2026 Jul 4;44(11):1948–57. doi: 10.1007/s11604-026-02041-2 (PMC13722725; doi:10.1007/s11604-026-02041-2)
Supplement: Supplementary file 1 [file 11604_2026_2041_MOESM1_ESM.docx]

***Atherosclerotic middle cerebral artery stenosis: adjacent arterial geometry, hemodynamics and plaque features***

[Supplemental Table 1. Patient and vessel geometry characteristics in those with proximal versus distal MCA-M1 plaques	2](#_Toc226904829)

[Supplemental Table 2. Patient and vessel geometry characteristics of those with proximal versus distal MCA-M1 plaques in moderate and severe stenosis subgroups	4](#_Toc226904830)

[Supplemental Table 3. The correlations of geometric characteristics with rWSS metrics in patients with proximal MCA-M1 plaques	7](#_Toc226904831)

[Supplemental Table 4. The correlations of geometric characteristics with rWSS metrics in patients with distal MCA-M1 plaques	9](#_Toc226904832)

[Supplemental Table 5. Interobserver agreement of geometric features measurements	11](#_Toc226904833)

# Supplemental Table 1. Patient and vessel geometry characteristics in those with proximal versus distal MCA-M1 plaques

| **Characteristics** | **Proximal MCA-M1 plaques (n=86)** | **Distal MCA-M1 plaques (n=46)** | **P value** |
| --- | --- | --- | --- |
| Age, year | 62 (53-71) | 60 (56-67) | 0.606 |
| Male | 56 (65.1) | 37 (80.4) | 0.066 |
| SBP at admission, mmHg | 155 (135-168) | 153 (134-178) | 0.809 |
| DBP at admission, mmHg | 83 (75-96) | 82 (78-95) | 0.802 |
| NIHSS | 2 (0-5) | 2 (0-4) | 0.991 |
| **Vascular risk factors** |  |  |  |
| Hypertension | 56 (65.1) | 27 (58.7) | 0.467 |
| Diabetes mellitus | 25 (29.1) | 16 (34.8) | 0.499 |
| Dyslipidemia | 48 (55.8) | 27 (58.7) | 0.750 |
| Smoking | 35 (40.7) | 21 (45.7) | 0.583 |
| Previous stroke/TIA | 13 (15.1) | 6 (13.0) | 0.746 |
| **Laboratory test results** |  |  |  |
| Fasting glucose, mmol/L | 5.5 (4.9-6.9) | 5.7 (5.2-7.8) | 0.193 |
| HbA1c, % | 5.8 (5.5-6.5) | 6.2 (5.7-7.5) | 0.095 |
| Triglyceride, mmol/L | 1.3 (1.0-1.9) | 1.4 (1.0-1.9) | 0.867 |
| HDL-C, mmol/L | 1.2 (1.0-1.4) | 1.1 (1.0-1.4) | 0.466 |
| LDL-C, mmol/L | 2.8 (2.2-3.6) | 3.0 (2.3-4.1) | 0.662 |
| **Vessel geometric characteristics** |  |  |  |
| Diameter of ICA terminal, mm | 3.60 (3.07-3.84) | 3.70 (3.35-3.99) | 0.098 |
| Diameter of ACA origin, mm | 2.34 (1.94-2.74) | 2.37 (1.96-2.67) | 0.890 |
| Diameter of MCA-M1 origin, mm | 2.60 (2.15-2.98) | 3.14 (2.65-3.39) | <0.001 |
| Diameter of MCA-M1 terminal, mm | 2.67 (2.27-2.88) | 2.49 (2.01-3.11) | 0.157 |
| Diameter ratio of MCA-M1 origin and terminal | 0.98 (0.83-1.17) | 1.19 (1.05-1.55) | <0.001 |
| The angle between ICA and ACA, ° | 121.19 (107.19-129.14) | 121.39 (111.46-128.07) | 0.860 |
| The angle between ICA and MCA-M1, ° | 54.22 (37.45-65.07) | 49.24 (38.14-56.68) | 0.305 |
| The angle between ACA and MCA-M1, ° | 139.96 (120.90-149.13) | 143.92 (128.95-152.70) | 0.561 |
| MCA-M1 tortuosity, % | 20.48 (10.81-35.60) | 13.64 (7.53-25.00) | 0.025 |

MCA-M1, M1 middle cerebral artery; SBP, systolic blood pressure; DBP, diastolic blood pressure; NIHSS, National Institute of Health Stroke Scale; TIA, transient ischemic attack; HbA1c, glycated hemoglobin; HDL-C, high-density lipoprotein cholesterol; LDL-C, low-density lipoprotein cholesterol; ICA, internal carotid artery; ACA, anterior cerebral artery

# Supplemental Table 2. Patient and vessel geometry characteristics of those with proximal versus distal MCA-M1 plaques in moderate and severe stenosis subgroups

| **Characteristics** | **Moderate stenosis (n=74)** | | **P value** | **Severe stenosis (n=58)** | | **P value** |
| --- | --- | --- | --- | --- | --- | --- |
|  | **Proximal MCA-M1 plaques (n=48)** | **Distal MCA-M1 plaques (n=26)** |  | **Proximal MCA-M1 plaques (n=38)** | **Distal MCA-M1 plaques (n=20)** |  |
| Age, year | 65 (53-74) | 63 (56-67) | 0.679 | 62 (52-67) | 60 (54-69) | 0.676 |
| Male | 29 (60.4) | 21 (80.8) | 0.076 | 27 (71.1) | 16 (80.0) | 0.463 |
| SBP at admission, mmHg | 159 (135-170) | 153 (135-169) | 0.865 | 157 (135-168) | 147 (125-178) | 0.533 |
| DBP at admission, mmHg | 89 (75-97) | 81 (69-87) | 0.153 | 80 (70-88) | 82 (78-100) | 0.055 |
| NIHSS | 3 (1-5) | 1 (0-4) | 0.295 | 1 (0-4) | 2 (1-3) | 0.110 |
| **Vascular risk factors** |  |  |  |  |  |  |
| Hypertension | 34 (70.8) | 15 (57.7) | 0.257 | 22 (57.9) | 12 (60.0) | 0.878 |
| Diabetes mellitus | 15 (31.3) | 9 (34.6) | 0.769 | 10 (26.3) | 7 (35.0) | 0.494 |
| Dyslipidemia | 27 (56.3) | 18 (69.2) | 0.275 | 21 (55.3) | 9 (45.0) | 0.457 |
| Smoking | 20 (41.7) | 10 (38.5) | 0.790 | 15 (39.5) | 11 (55.0) | 0.263 |
| Previous stroke/TIA | 9 (18.8) | 3 (11.5) | 0.425 | 4 (10.5) | 3 (15.0) | 0.622 |
| **Laboratory test results** |  |  |  |  |  |  |
| Fasting glucose, mmol/L | 5.6 (5.0-7.0) | 5.9 (5.2-6.9) | 0.608 | 5.0 (4.7-5.8) | 5.7 (4.7-9.2) | 0.193 |
| HbA1c, % | 6.0 (5.6-6.8) | 6.3 (5.8-7.2) | 0.132 | 5.6 (5.4-6.2) | 6.1 (5.6-7.5) | 0.434 |
| Triglyceride, mmol/L | 1.3 (0.9-1.9) | 1.4 (1.1-1.6) | 0.488 | 1.2 (1.1-1.9) | 1.3 (0.8-2.2) | 0.778 |
| HDL-C, mmol/L | 1.2 (1.0-1.4) | 1.2 (0.9-1.4) | 0.176 | 1.1 (1.0-1.4) | 1.1 (1.0-1.3) | 0.871 |
| LDL-C, mmol/L | 2.7 (2.2-3.9) | 3.3 (2.4-4.6) | 0.176 | 3.0 (2.1-3.6) | 2.5 (1.9-3.3) | 0.415 |
| **Geometric characteristics** |  |  |  |  |  |  |
| Diameter of ICA terminal, mm | 3.60 (3.07-3.86) | 3.84 (3.38-4.08) | 0.193 | 3.62 (3.09-3.83) | 3.64 (3.06-3.91) | 0.295 |
| Diameter of ACA origin, mm | 2.32 (2.02-2.67) | 2.33 (2.03-2.74) | 0.923 | 2.49 (1.94-2.92) | 2.38 (1.91-2.61) | 0.980 |
| Diameter of MCA-M1 origin, mm | 2.60 (2.20-2.82) | 3.15 (2.83-3.42) | <0.001 | 2.78 (1.96-3.03) | 2.96 (2.56-3.27) | 0.022 |
| Diameter of MCA-M1 terminal, mm | 2.73 (2.37-3.01) | 2.59 (2.19-3.07) | 0.300 | 2.63 (2.18-2.82) | 2.20 (1.71-3.21) | 0.199 |
| Diameter Ratio of MCA-M1 origin and terminal | 0.90 (0.83-1.05) | 1.16 (1.11-1.30) | <0.001 | 1.06 (0.75-1.28) | 1.21 (0.94-1.66) | 0.008 |
| The angle between ICA and ACA, ° | 113.61 (103.57-127.97) | 123.48 (111.47-126.33) | 0.213 | 122.98 (112.15-129.77) | 120.02 (105.60-129.70) | 0.280 |
| The angle between ICA and MCA-M1, ° | 55.26 (27.99-65.30) | 47.28 (37.70-52.20) | 0.647 | 56.42 (41.46-65.93) | 51.42 (39.85-61.88) | 0.326 |
| The angle between ACA and MCA-M1, ° | 134.28 (119.52-147.73) | 145.01 (130.23-153.33) | 0.199 | 142.09 (129.47-151.90) | 140.37 (118.16-147.02) | 0.513 |
| MCA-M1 tortuosity, % | 20.48 (11.11-42.86) | 19.05 (9.89-28.21) | 0.504 | 23.46 (9.89-33.33) | 11.11 (7.53-19.05) | 0.013 |

MCA-M1, M1 middle cerebral artery; SBP, systolic blood pressure; DBP, diastolic blood pressure; NIHSS, National Institute of Health Stroke Scale; TIA, transient ischemic attack; HbA1c, glycated hemoglobin; HDL-C, high-density lipoprotein cholesterol; LDL-C, low-density lipoprotein cholesterol; ICA, internal carotid artery; ACA, anterior cerebral artery

# Supplemental Table 3. The correlations of geometric characteristics with rWSS metrics in patients with proximal MCA-M1 plaques

|  | **Diameter of ICA terminal, mm** | **Diameter of ACA origin, mm** | **Diameter of MCA-M1 origin, mm** | **Diameter of MCA-M1 terminal, mm** | **Diameter ratio of MCA-M1 origin and terminal** | **The angle between ICA and ACA, °** | **The angle between ICA and MCA-M1, °** | **The angle between ACA and MCA-M1, °** | **MCA-M1 tortuosity** |
| --- | --- | --- | --- | --- | --- | --- | --- | --- | --- |
| **Throughout the MCA-M1 plaque** | | | | | | | | | |
|  | **Spearman R** | **Spearman R** | **Spearman R** | **Spearman R** | **Spearman R** | **Spearman R** | **Spearman R** | **Spearman R** | **Spearman R** |
| **Maximum rWSS** | -0.144 | 0.018 | -0.273^*^ | -0.171 | -0.137 | -0.001 | 0.236^*^ | -0.009 | 0.002 |
| **Area of high-rWSS region, mm^2^** | -0.125 | 0.077 | -0.319^**^ | -0.067 | -0.273^*^ | 0.058 | 0.330^**^ | 0.008 | -0.155 |
| **Mean rWSS of high-rWSS region** | -0.203 | -0.031 | -0.300^**^ | -0.218^*^ | -0.132 | 0.001 | 0.237^*^ | 0.002 | 0.031 |
| **Upstream MAC-M1 plaque segment** | | | | | | | | | |
|  | **Spearman R** | **Spearman R** | **Spearman R** | **Spearman R** | **Spearman R** | **Spearman R** | **Spearman R** | **Spearman R** | **Spearman R** |
| **Maximum rWSS** | -0.142 | 0.002 | -0.291^**^ | -0.147 | -0.186 | -0.028 | 0.270^*^ | -0.052 | 0.011 |
| **Area of high-rWSS region, mm^2^** | -0.143 | 0.052 | -0.459^**^ | -0.002 | -0.465^**^ | -0.016 | 0.379^**^ | -0.045 | -0.150 |
| **Mean rWSS of high-rWSS region** | -0.197 | 0.017 | -0.384^**^ | -0.161 | -0.260^*^ | -0.026 | 0.308^**^ | 0.033 | 0.008 |
| **Downstream MCA-M1 plaque segment** | | | | | | | | | |
|  | **Spearman R** | **Spearman R** | **Spearman R** | **Spearman R** | **Spearman R** | **Spearman R** | **Spearman R** | **Spearman R** | **Spearman R** |
| **Maximum rWSS** | -0.156 | 0.024 | -0.299^**^ | -0.177 | -0.153 | -0.004 | 0.217^*^ | 0.013 | 0.003 |
| **Area of high-rWSS region, mm^2^** | -0.103 | 0.084 | -0.078 | -0.086 | 0.023 | 0.048 | 0.291^**^ | 0.076 | -0.059 |
| **Mean rWSS of high-rWSS region** | -0.161 | 0.021 | -0.222^*^ | -0.133 | -0.073 | -0.030 | 0.238^*^ | -0.038 | 0.074 |

*p < 0.05; ** p < 0.01

rWSS, relative wall shear stress; MCA-M1, M1 middle cerebral artery; ICA, internal carotid artery; ACA, anterior cerebral artery

# Supplemental Table 4. The correlations of geometric characteristics with rWSS metrics in patients with distal MCA-M1 plaques

|  | **Diameter of ICA terminal, mm** | **Diameter of ACA origin, mm** | **Diameter of MCA-M1 origin, mm** | **Diameter of MCA-M1 terminal, mm** | **Diameter ratio of MCA-M1 origin and terminal** | **The angle between ICA and ACA, °** | **The angle between ICA and MCA-M1, °** | **The angle between ACA and MCA-M1, °** | **MCA-M1 tortuosity** |
| --- | --- | --- | --- | --- | --- | --- | --- | --- | --- |
| **Throughout the MCA-M1 plaque** | | | | | | | | | |
|  | **Spearman R** | **Spearman R** | **Spearman R** | **Spearman R** | **Spearman R** | **Spearman R** | **Spearman R** | **Spearman R** | **Spearman R** |
| **Maximum rWSS** | -0.260 | -0.039 | -0.215 | -0.156 | 0.001 | -0.077 | -0.017 | 0.086 | 0.064 |
| **Area of high-rWSS region, mm^2^** | -0.222 | -0.083 | -0.176 | -0.121 | -0.073 | -0.144 | 0.090 | 0.242 | 0.056 |
| **Mean rWSS of high-rWSS region** | -0.200 | 0.011 | -0.267 | -0.199 | 0.061 | -0.044 | -0.048 | 0.091 | 0.015 |
| **Upstream MCA-M1 plaque segment** | | | | | | | | | |
|  | **Spearman R** | **Spearman R** | **Spearman R** | **Spearman R** | **Spearman R** | **Spearman R** | **Spearman R** | **Spearman R** | **Spearman R** |
| **Maximum rWSS** | -0.161 | -0.024 | -0.127 | -0.077 | -0.025 | -0.121 | 0.017 | -0.007 | 0.093 |
| **Area of high-rWSS region, mm^2^** | -0.044 | -0.016 | -0.203 | 0.009 | -0.156 | -0.160 | 0.176 | 0.038 | 0.074 |
| **Mean rWSS of high-rWSS region** | -0.199 | -0.064 | -0.153 | -0.136 | 0.005 | -0.136 | -0.007 | -0.030 | 0.012 |
| **Downstream MCA-M1 plaque segment** | | | | | | | | | |
|  | **Spearman R** | **Spearman R** | **Spearman R** | **Spearman R** | **Spearman R** | **Spearman R** | **Spearman R** | **Spearman R** | **Spearman R** |
| **Maximum rWSS** | -0.263 | -0.017 | -0.261 | -0.198 | 0.010 | -0.069 | 0.001 | 0.124 | 0.047 |
| **Area of high-rWSS region, mm^2^** | -0.265 | -0.006 | -0.187 | -0.353^*^ | 0.154 | -0.121 | 0.078 | 0.277 | -0.066 |
| **Mean rWSS of high-rWSS region** | -0.193 | 0.001 | -0.206 | -0.206 | 0.043 | 0.022 | -0.018 | 0.110 | -0.009 |

*p < 0.05

rWSS, relative wall shear stress; MCA-M1, M1 middle cerebral artery; ICA, internal carotid artery; ACA, anterior cerebral artery

# Supplemental Table 5. Interobserver agreement of geometric features measurements

| **Geometric features** | Interobserver agreement  Kappa (p) or ICC (95% CI) |
| --- | --- |
| **Diameter of ICA terminal** | 0.968 (0.910–0.978) |
| **Diameter of ACA origin** | 0.962 (0.858–0.984) |
| **Diameter of MCA-M1 origin** | 0.974 (0.918–0.987) |
| **Diameter of MCA-M1 terminal** | 0.988 (0.961–0.994) |
| **The angle between ICA and ACA** | 0.984 (0.837–0.993) |
| **The angle between ICA and MCA-M1** | 0.983 (0.924–0.993) |
| **The angle between ACA and MCA-M1** | 0.989 (0.951–0.994) |
| **MCA-M1 tortuosity** | 0.912 (0.686-0.977) |
| **Proximal- and distal-plaque** | 0.737 (0.016) |

ICA, internal carotid artery; ACA, anterior cerebral artery; MCA-M1, M1 middle cerebral artery; ICC, intraclass correlation coefficient.
